# Microbial modulation of plant ethylene signaling: ecological and evolutionary consequences

**DOI:** 10.1186/s40168-018-0436-1

**Published:** 2018-03-21

**Authors:** Mohammadhossein Ravanbakhsh, Rashmi Sasidharan, Laurentius A. C. J. Voesenek, George A. Kowalchuk, Alexandre Jousset

**Affiliations:** 1 Ecology and Biodiversity, Institute of Environmental Biology, Utrecht University, 3584 CH Utrecht, The Netherlands; 2 Plant Ecophysiology, Institute of Environmental Biology, Utrecht University, 3584 CH Utrecht, The Netherlands

**Keywords:** Evolution, Holobiont, Plant, Phenotype, Physiology, Ethylene, Microbiota, Microbiome, ACC deaminase

## Abstract

The plant hormone ethylene is one of the central regulators of plant development and stress resistance. Optimal ethylene signaling is essential for plant fitness and is under strong selection pressure. Plants upregulate ethylene production in response to stress, and this hormone triggers defense mechanisms. Due to the pleiotropic effects of ethylene, adjusting stress responses to maximize resistance, while minimizing costs, is a central determinant of plant fitness. Ethylene signaling is influenced by the plant-associated microbiome. We therefore argue that the regulation, physiology, and evolution of the ethylene signaling can best be viewed as the interactive result of plant genotype and associated microbiota. In this article, we summarize the current knowledge on ethylene signaling and recapitulate the multiple ways microorganisms interfere with it. We present ethylene signaling as a model system for holobiont-level evolution of plant phenotype: this cascade is tractable, extremely well studied from both a plant and a microbial perspective, and regulates fundamental components of plant life history. We finally discuss the potential impacts of ethylene modulation microorganisms on plant ecology and evolution. We assert that ethylene signaling cannot be fully appreciated without considering microbiota as integral regulatory actors, and we more generally suggest that plant ecophysiology and evolution can only be fully understood in the light of plant-microbiome interactions.

## Background

### Environmental stress and plant fitness

Plants are constantly facing a range of different environmental stressors linked for instance to temperature, water availability, presence of toxic minerals, or pathogens. Stress can be permanent, for instance, when a plant lives outside its ecological optimum, or acute during climatic extremes such as drought and flooding waves. Environmental stress has an important effect on plant fitness and elicits specific adaptations [1]. Plants have evolved a range of physiological and morphological responses to stressors, allowing them to cope with the prevailing environmental conditions. Although these responses vary widely, they all share one characteristic: they all come at a cost to the plant, diverting resources from growth and reproduction, and causing negative side effects that may have consequences on other traits that can result in indirect fitness costs. Optimizing the relative investment into stress response and other life history traits is thus essential to maximize fitness [2]. Due to the variability of stressors and their interactive effects with plant genotype, regulating stress response is a complex task with several possible optima. Further, adaptation to one specific set of environmental conditions may negatively affect plant fitness under other conditions [3]. Plant transpiration illustrates this dilemma well: stomatal closure, a typical plant response to drought, reduces water loss, but comes at a cost of lower photosynthesis, gas exchange and sap flow. Given that stomata are also an entry point for several pathogens [4], the optimal aperture will be a function of several parameters including water availability, plant sensitivity to desiccation, and presence of pathogens [5].

To optimize fitness, stress responses must be therefore carefully adjusted. This implies that plants need to perceive different stressors, process the signals, and trigger an optimal stress response, thereby maximizing resistance while minimizing costs and side effects. Signal integration in plants is generally achieved by alterations in hormonal balance. Hormones such as ethylene, auxin, or jasmonic acid interactively shape the relative investment of plants into growth, reproduction, and stress defense [6]. Hormone concentrations are dictated by the combined action of the plant’s own regulatory pathways as well as the activities of its associated microbiota. Hormonal regulation therefore offers an excellent model to approach plant physiology, ecology, and evolution from a holobiont perspective in which plants and microbes form a coherent unit of selection [7].

In this review, we approach ethylene, a central plant hormone regulating the balance between growth and stress tolerance, from a holobiont perspective. We first briefly, summarize the importance of ethylene for stress tolerance and other life history traits. We then go on to provide an overview of how plants and their associated microbiota jointly shape hormonal balances, thereby shifting plant response toward or away from adaptation to specific situations.

## Regulation of stress response by plants

### Role of ethylene

Ethylene is a central plant hormone regulating several aspects of plant growth and development, throughout the whole plant life cycle, from germination to senescence [8]. In addition, this hormone is essential to regulate stress responses and confer stress tolerance [9, 10]. Stress results in increased levels of ethylene in plants. Stress-derived ethylene is a signal triggering adaptive responses and influences other hormonal signaling pathways [11]. Due to the multiple effects of ethylene on plant phenotype, increased ethylene levels will induce a range of pleiotropic effects, such as growth inhibition and late flowering [12], in addition to the target response [13]. Precisely controlling cellular ethylene levels is thus a key aspect of plant physiology [13].

### Ethylene production, signal transduction, and response

**Fig. 1:**
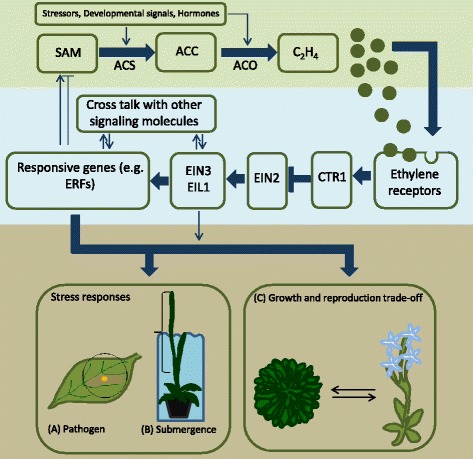
Overview of the pathways linked to ethylene production (top panel), signal transduction (central panel), and response (bottom panel). Ethylene concentration determines plant resource allocation into growth, reproduction, and stress response [13]. The thick arrows show the main ethylene cascade, and the thin ones point to possible interaction with external and internal stimuli. We illustrate plant response with three well-investigated ethylene-dependent phenotypic adaptations. **a** Ethylene coordinates plant response against pathogens, such as hypersensitive response, preventing pathogen spread [20]. **b** Ethylene accumulation triggers escape strategy involving accelerated shoot growth in submerged plants, allowing them to regain atmospheric contact [82]. **c** Growth-reproduction tradeoffs: higher ethylene causes plants to invest more resources into seed production under harsh conditions that may compromise vegetative stage survival. SAM *S*-adenosylmethionine, ACC 1-aminocyclopropane-1-carboxylic acid, ACS ACC synthase, ACO ACC oxidase, C2H4 plant hormone ethylene, CTR1 constitutive triple response 1, EIN2 ethylene-insensitive protein 2, EIN3 ethylene-insensitive protein 3, EIL1 ethylene insensitive 3-like 1 protein, ERFs ethylene response factors

An important step in ethylene production is the synthesis of its precursor ACC (1-aminocyclopropane-1 carboxylic acid) by ACC synthase (ACS) enzyme (Fig. 1). Upon stress detection, ACS mediates the synthesis of the ethylene precursor ACC, which is transformed to ethylene by the enzyme ACC oxidase (ACO; Fig. 1). Both *ACS* and *ACO* form large multigene families in plants and different members can be regulated by different internal and external stimuli [14–16]. Ethylene binding to its receptors triggers the expression of downstream response genes. In the absence of ethylene, the ethylene receptors activate CTR1 which is a negative regulator of ethylene signalling. Ethylene binding inactivates the receptors and therefore CTR1. This consequently relieves the inhibition of EIN2, a positive regulator of ethylene signaling. Downstream of EIN2 are the transcription factors EIN3 (ethylene-insensitive 3) and its homolog EIL1 (ethylene-insensitive 3-like 1) that are primary mediators of the transcriptional responses to ethylene [17, 18]. These transcription factors will increase the expression of ethylene responsive transcription factors (ERFs) [19], resulting in ethylene-mediated stress responses in plants [20]. ERF-regulated traits include activation of plant immunity [21, 22], metabolic and morphological adaptations to flooding [9, 23], expression of systems for scavenging reactive oxygen species and modification of enzymatic activity under heavy metal and salinity stress conditions [24–26]. The type of ethylene-mediated response is highly variable, as discussed below in “ethylene variation in plants.”

### Ethylene varies as a function of stress type and intensity

Ethylene production depends on the intensity and duration of stress periods. For instance, different levels of heavy metal [25, 27] or different dehydration rates [28] differentially modulate ethylene biosynthesis and signaling. In another example, low-levels of stress stimulate ethylene production, while high levels may decrease it [28, 29], either as part of a targeted stress response or as the result of impaired plant metabolism.

### Ethylene response from ecological and agricultural perspective

**Fig. 2:**
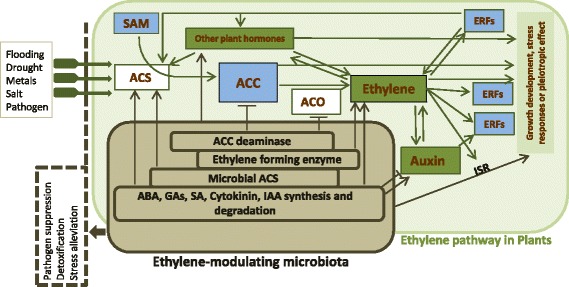
A holobiont-level regulation of ethylene signaling and plant stress response. Ethylene pathway in plants (green area). ACC (1-aminocyclopropane-1-carboxylic acid) is synthesized from SAM (*S*-adenosylmethionine) by the action of ACC synthase enzyme (ACS). ACC is then converted to ethylene by the enzyme ACC oxidase (ACO), triggering different ethylene response factors (ERFs). Plant-associated microorganisms can alter virtually all steps of ethylene signaling. Some species can increase ethylene levels by producing ACC oxidase (microbial ethylene-forming enzyme), by inducing ACC synthase in plant or by affecting other plant hormones indirectly. They can also modulate ethylene response by producing plant hormones that interact with ethylene signaling [62, 83, 84]. Other microorganisms can also decrease ethylene production by cleaving its precursor ACC. White boxes show ethylene biosynthetic enzymes, green boxes show plant hormones and signals, and blue boxes show the molecules involved in the ethylene pathway. ABA abscisic acid, GA gibberellic acid, SA salicylic acid

Plants are continually confronted with variable environmental conditions, to which they must respond and adapt. Ethylene has a central role in plant survival and adaptation in dynamic environments. Ethylene-dependent stress response enhances survival in stress conditions such as heavy metal [25], salinity [26], and drought [30]. However, high stress tolerance may cause pleiotropic effects on plant phenotype under such stress conditions [25]. Ethylene triggers for instance early flowering, helping plants complete their life cycle before resources become depleted [30]. This comes, however, at the cost of a reduced biomass [13]. Therefore, considering multiple components of plant fitness may be essential to provide a full view of ethylene-related impacts. Instead of purely looking at single traits such as short-term biomass production, as has often been done in plant-microbe research, we propose approaching ethylene as coordinator balancing different life history traits to reach the best possible phenotype for survival in prevailing ambient conditions, thereby maximizing reproductive fitness. Variation in ethylene levels might be due to selection on the plant genetic material or, as we will discuss in the next section, on the microorganisms that co-regulate ethylene (Fig. 2).

## Ethylene variation in plants

### Variability in ethylene-based stress responses across plant species

The core ethylene transduction cascade is highly conserved in plants, and a wide range of plants use ethylene as a regulator of their stress responses. However, plants vary greatly in how ethylene impacts stress perception, transduction, and the final response. Plants evolved in a certain environment have adapted ethylene signaling to the stresses typical for that environment or even dropped it, if not useful. For instance, plants living in flood-prone or riparian areas, including rice and *Rumex palustris*, use flooding-induced accumulation of ethylene to trigger important adaptive responses [31]. In contrast, some aquatic plants adapted to a permanently submerged lifestyle and lost many genes involved in ethylene signaling [32, 33]. Contrasting ethylene-mediated responses are even seen in closely related plant species. For instance, different species of *Rumex* sp. or different varieties of rice all use ethylene as a flooding signal to trigger adaptive responses, yet the responses itself are highly variable, ranging from compensatory growth to complete quiescence [31, 34].

### Variability in stress perception and ethylene signal transduction

Both *ACS* and *ACO* ethylene biosynthetic genes are encoded by large multigene families. These genes are organ-specific and are differentially regulated by different environmental signals [15, 35, 36]. The size of these multigene families can vary between plant species and could link to the variation in ethylene-mediated stress perception. For instance, apple harbors 19 *ACS* genes as compared to 12 *ACS* genes in *Arabidopsis thaliana* and 9 in tomato [36–38]. Deletion of one single gene, *ACS6*, resulted for instance in a 85–90% reduction in ethylene production in maize [39]. Variation in the ethylene response can also occur at the level of ethylene perception across different plant genotypes linked to changes in receptor affinity, expression pattern, and/or turn-over [40]. Furthermore, knocking out *EIN3* showed opposite effects on salinity tolerance in *Arabidopsis* and rice [41]. Ethylene signaling and response are highly dependent on plant genotype [42], organ, growth stage [43], and associated microbiota.

## Microbiota

### Importance of microbiota as co-regulator of stress response

Plants are associated with a complex microbiome, including bacteria, fungi, and protists that have impact on diverse aspects of plant growth, health, and evolution [44]. Plant-associated microbiota can be either vertically transmitted, as is the case for endophytes that live within plant tissues, or horizontally for instance by recruiting microbiota to the rhizosphere from the surrounding soil species pool. Microbiota form an integral part of a plant’s immune system, metabolism, and hormonal balance [45]. They directly alleviate stress, for instance, by producing protective compounds that enhance drought resistance [34, 46], by degrading organic pollutants [47], or by chelating heavy metals [48]. Plant-associated microbes can also fine-tune hormonal balance and physiology by modulated plant hormone levels and the pathways they steer. In the case of ethylene, several possible mechanisms have been described by which microorganisms can affect plant hormonal levels. Below, we examine the mechanisms by which the plant-microbe dialog determines ethylene-mediated plant responses as the basis for a more general model on holobiont-level regulation of plant hormonal balance.

### Ethylene modulation as a holobiome process

Ethylene signaling forms a perfect example of a holobiont-level physiological cascade. From the holobiont perspective, plant physiology is controlled by a combination of traits encoded in the host genome as well as its associated microbes, which collectively form the holobiont [7]. This association offers a broader genetic pool than the plant alone: ethylene-modulating microbes could increase the reservoir of genetic information linked to ethylene signaling, enabling a greater plant phenotypic plasticity in response to stressors. The microbiota can (1) impact plant-perceived stress, (2) co-regulate ethylene which affects plant fitness, and (3) perceive ethylene, potentially responding to it.

### Plant and ethylene-modulating microbes as unit of selection

Ethylene levels are a strong determinant of fitness in dynamic environments. Given that plants and microbes work in concert to modulate ethylene-mediated responses, the holobiont level of selection is the most appropriate: the ethylene cascade provides an important link between the host and its associated microbes, and forms an integrated biological entity [44]. This interaction even has the potential to be evolutionarily stable: plants rely on microbes to optimize their fitness and microbes directly benefit from a more vigorous host that may provide more nutrients and energy. As plants can select associated microbes on the basis of the functions they perform, mutualistic interactions may persist across generations.

## Ethylene modulation by microorganisms: evolutionary impact on plants

- Reduction of stress perception by microorganisms

**Fig. 3:**
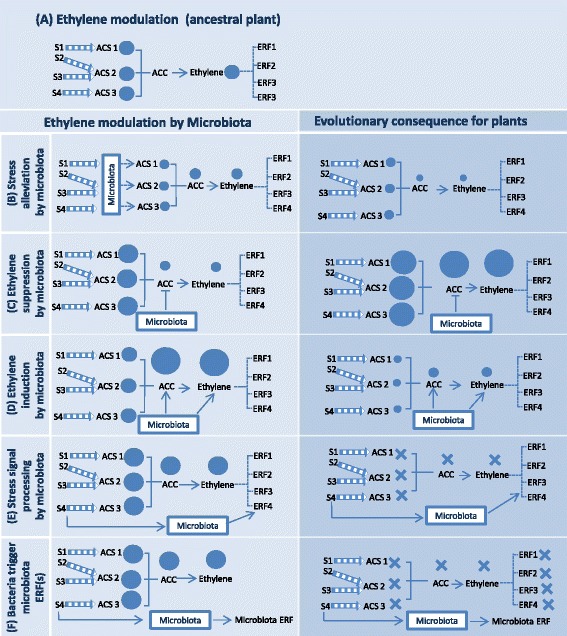
**a** Potential consequences of evolution of an intertwined ethylene signaling involving both plant and microbiota. In ancestral plant phenotype, ACC (1-aminocyclopropane-1-carboxylic acid) is produced by the action of ACC synthase enzymes (ACS). ACC is then converted to ethylene by the enzyme ACC oxidase, triggering different ethylene response factors (ERFs). **b** Bacteria reduce the intensity of stress experienced by the plant. Plant reliance on the microbiome to reduce stressors may lead to a reduced ability of the plant to respond to the acute stressors. **c**, **d** Bacteria alter ACC and ethylene in plants, leading to over- or under-expression of ethylene pathway genes in plants. **e**, **f** Microorganisms integrate plant signals and trigger plant ethylene response factors (ERFs) or express their own ERFs, contributing to partial or complete loss of ethylene pathway in the plant. The dashed lines (for instance, between stressors and ACS and ethylene and ERFs) showed indirect connections. The size of each circle indicates relative levels of ACC synthase (ACS) activity, ACC, and ethylene production in response to stressors (S1–S4)

Microorganisms may contribute to plant stress tolerance in an ethylene-independent way by providing protection mechanisms expressed outside of the host plant. For instance, plant-associated microbiota may reduce the intensity of stress experienced by the plant by detoxifying chemicals or providing protective substances against desiccation [46–48]. From an evolutionary perspective, a plant’s reliance on the microbiome to reduce stressors may lead to a reduced ability of the plant to respond to the acute stressors (Fig. 3b), a task delegated to the associated microbiota.

b) Alteration of ethylene level by microorganisms

Microbes can potentially influence all regulatory steps of the ethylene pathway (Fig. 2). The most direct way of acting on ethylene signaling is to either directly produce or degrade ethylene. Several plant-associated microbes can increase plant ethylene levels by directly synthesizing ethylene or inducing plant ACS activity [49–52]. Ethylene production by microbes was first reported in the pathogen *Ralstonia solanacearum*, which, among other symptoms, induces banana premature ripening [52]. Microbial ethylene production was later mainly investigated in relation to pathogenic bacteria [50, 51]. However, biosynthetic pathway studies [53] and the examination of available bacterial genomes have revealed that the relevant genes and pathways can be found across a wide range of microorganisms [54, 55]. For instance, more than one third of all cultivable soil bacteria can produce ethylene via different pathways [53]. Phylogenetic studies of ethylene-forming enzymes show that multiple ethylene-producing pathways have evolved independently and later spread between bacterial phyla by horizontal gene transfer [53, 56]. Ethylene production by microbes may have deep effects on plant physiology and life history, as demonstrated by the accelerated fruit ripening in plants inoculated with *Escherichia coli* engineered to produce ACC oxidase [57]. Rhizosphere microbes can further increase ethylene indirectly by secreting auxin [58, 59] and cytokinin [58, 60], two hormones that upregulate the expression of ACS-coding genes [61, 62].

Microbioorganisms can also decrease ethylene levels, for instance, by producing ACC deaminase. This enzyme degrades ACC, ultimately leading to lower plant ethylene concentrations. ACC deaminase can be found in both commensal [63] and pathogenic microbes [64]. ACC deaminase genes are widespread in bacteria, fungi, and members of stramenopiles [65]. ACC deaminase genes of bacteria and fungi shared high sequence identity [65], pointing to a single evolutionary origin and frequent horizontal transfer of this gene in bacteria and fungi [65–67]. The reduction of ethylene levels caused by ACC-deaminase producing microbes is of the same magnitude as the one resulting from knocking out *ACS* genes [39, 68]. In contrast to commonly held assumptions, ACC deaminase-producing microbes are not necessarily good and the effects of ACC deaminase on plant physiology and plant growth greatly depend on the interactive effects of plant genotype [69, 70] and the environment [68, 71]. For instance, root growth reduction by ethylene is a common adaptation to avoid salt and pollutants [72]. Alleviating this inhibition may bring a short-term increase in root growth, but may ultimately be deleterious for the plant.

Co-evolution of plants with bacteria that increase or inhibit ethylene levels may have various consequences: co-evolution of plants with microbes increasing ethylene levels may cause the plant to reduce ethylene production in order to maintain homeostasis. This could result in a lower ability to produce ethylene, a higher sensitivity to stressors and a dependency on microbial ethylene production (Fig. 3d). In contrast, plants associated with ethylene-reducing microorganisms such as ACC deaminase producers may need to produce more ACC to compensate for microbial degradation (Fig. 3c). Thus, plants may evolve a higher expression of *ACS* genes, which can not only allow a wide range of responses, but may also lead to overreactions to stress without modulation by the associated microbiome.

### Microbial alteration of plant ethylene response and intertwined signaling

In addition to direct manipulation of ethylene levels, the microbiota is an integral component of stress perception and response. For instance, some microbial species can perceive environmental stressors relevant to the plant [73] as well as sense and respond to plant ethylene [32]. This suggests that they may potentially be part of the holobiont-level ethylene-regulated traits, communicating stress perception to plants, and monitoring plant stress status. As ethylene sensitivity in plant-associated microbiota is widespread, we propose that ethylene signaling may be part of a hologenome-level stress response in which genetic traits carried by both microbiota and the plant are activated in response to stressors. Ethylene modulation is under this perspective the result of the co-evolution of both plant and microbial traits. Bacteria could receive signals from environment or plants and trigger plant ethylene response factors (Fig. 3e) or even express their own ethylene response factors in response to plant ethylene or environmental cues (Fig. 3f).

Such intertwined signaling between plants and microbes might contribute to complete [74] or a partial [75] loss of plant ethylene pathways over the course of evolution, as observed in the loss of the ACC biosynthetic route in several gymnosperms [76] or the production of ethylene via an ACC-independent pathway in several plants [75]. From a co-evolutionary perspective, such plants will become more dependent on microbiota for ethylene pathway modulation.

## Box 1

### What makes co-evolution possible?

- Plants live in close association with a wide range of microbes. Roots select and feed a specific microbiome [77]. A wide range of the rhizosphere-enriched microorganisms have the ability to modulate plant ethylene signaling. For instance, genes linked to ethylene production or reduction can be found in a broad range of bacteria and fungi [65, 78, 79]. The constant contact with an ethylene signaling-altering microbiota may cause the evolution of a modified pathway optimizing plant response in the presence of external perturbations.
- Positive feedback loops: under stressful conditions, plants produce more ACC [14]. This confers an advantage to microorganisms producing ACC deaminases that are able to use ACC as a nitrogen and carbon source. This may result in an increased density of ACC-degrading microbes, whose effect can be counteracted by the plant by producing more ACC. The outcome might be beneficial only for microbes (parasitism of plant nitrogen), or mutually beneficial (symbiosis via shared ethylene signaling).
- Plant adaptation to fluctuating environments requires a rapid rewiring of stress response pathways such as ethylene signaling. However, this adaptation may be too slow in plants, requiring several generations to acquire and spread the needed mutations. Emergence of genetic variation in the microbiome is many orders of magnitude faster than in plants [80]. Modulation of plant hormone levels via the microbiome may thus provide a new mechanism to match plant phenotype to environmental conditions.
- Modulation of plant hormone levels via the microbiome may thus provide a new mechanism to match plant phenotype to environmental conditions.
- Ethylene-modulating microorganisms can be transmitted vertically, from one generation to the next generation, thus allowing co-evolution of microbes and the host as a cohesive unit of selection [80]. Vertical co-evolution may allow more gene transfer to the next generation, and the establishment of relatively stable associations. Nonetheless, vertical transmission is probably essential for the last of our proposed co-evolutionary dynamics (intertwined signaling; Fig. 3e, f). The ethylene modulation genes could transfer between ethylene-modulating bacteria by horizontal transfer [66, 67] and through symbiotic island exchange [81].

### Evolutionary implications of ethylene manipulation by plant-associated microorganisms

Based on existing scientific evidence, ethylene signaling most likely evolved within the context of long-term co-evolution processes between plants and their associated microbes. We propose that the joint regulation between microbes and plants can lead to several implications:

- Alterations of ethylene signaling may offer new functions and shift the niche of the holobiont
  Altering ethylene levels might allow plants to exploit new niche space, where other trait combinations are optimal. Co-evolution leading to ethylene overproduction (Fig. 3c) and insensitivity (Fig. 3d) might also shift plant niches, as well as restrict the chances for a plant to re-inhabit its ancestral range.
- Change in plant-encoded ethylene signaling genes
  During co-evolution, some microorganisms are potentially part of holobiont-level ethylene-regulated traits (Fig. 3e, f). This association might reduce some parts of the plant genome working in parallel with the microbiota, saving the cost of gene expression and maintenance of redundant genes. In addition to losing some part of the ethylene signaling pathway, based on the amount of plant dependency on associated microbes, dispersal of seeds to new environments with completely different microbial communities might cause them to die before they are able to adapt to the new conditions and pass the traits down to their offspring.
- Uncoupling plant phenotype from mutations in the plant genome
  Mutations can alter plant evolution by affecting different pathways including the ethylene pathway. Mutations in the plant ethylene biosynthesis and signaling pathway (for instance, the ability to overproduce ethylene) could cause new morphological traits or functions that promote plant fitness in a new environment, and therefore increase the chances for natural selection. Associated microbiota influence this selection by making the ancestral microbe-associated plants more successful in competition, thereby decreasing the advantage of mutations, as microbes might override the plant-bacteria co-evolution by altering different parts of the ethylene pathway.

## Conclusion

The plant hormone ethylene mediates many aspects of plant life history. At the holobiont level, ethylene signaling is a regulatory cascade composed of both plant- and microbiota-associated traits, which together provide a dynamic and fine-tuned response to environmental conditions and stressors. The holobiont perspective in plant hormone regulation also has large evolutionary implications in which plants have become dependent on their microbiome for fully adaptive ethylene-mediated responses. From an agricultural perspective, the plant holobiont may facilitate appropriate or maladapted stress responses depending on the match or mismatch of plant and microbiome traits. Many aspects of plant health related to the microbiome and ethylene signaling may represent a useful model case to further our general understanding of plant holobiont ecology and evolution.
